# CD8^+^ T Cells in SARS-CoV-2 Induced Disease and Cancer—Clinical Perspectives

**DOI:** 10.3389/fimmu.2022.864298

**Published:** 2022-04-01

**Authors:** Keywan Mortezaee, Jamal Majidpoor

**Affiliations:** ^1^Department of Anatomy, School of Medicine, Kurdistan University of Medical Sciences, Sanandaj, Iran; ^2^Department of Anatomy, Faculty of Medicine, Infectious Diseases Research Center, Gonabad University of Medical Sciences, Gonabad, Iran

**Keywords:** CD8^+^ T cell, SARS-CoV-2, cancer, exhaustion, memory T cell, immune checkpoint inhibitor (ICI), hypoxia, programmed death 1 receptor (PD-1)

## Abstract

Dysregulated innate and adaptive immunity is a sign of SARS-CoV-2-induced disease and cancer. CD8^+^ T cells are important cells of the immune system. The cells belong to the adaptive immunity and take a front-line defense against viral infections and cancer. Extreme CD8^+^ T-cell activities in the lung of patients with a SARS-CoV-2-induced disease and within the tumor microenvironment (TME) will change their functionality into exhausted state and undergo apoptosis. Such diminished immunity will put cancer cases at a high-risk group for SARS-CoV-2-induced disease, rendering viral sepsis and a more severe condition which will finally cause a higher rate of mortality. Recovering responses from CD8^+^ T cells is a purpose of vaccination against SARS-CoV-2. The aim of this review is to discuss the CD8^+^ T cellular state in SARS-CoV-2-induced disease and in cancer and to present some strategies for recovering the functionality of these critical cells.

## Highlights

• CD8^+^ T cells are infrequent in severe SARS-CoV-2-induced diseases.

• Vaccination against SARS-CoV-2 induces CD8^+^ T-cell responses.

• Vascular abnormality-related CD8^+^ T-cell dysfunction is potentiated in SARS-CoV-2 cancer cases.

• Stem-like and bystander CD8^+^ T cells strengthen defense against SARS-CoV-2 and cancer.

• Immunological competence is higher in antiviral vs. antitumoral CD8^+^ T cells.

• Aging, obesity, hypoxia, and chronic inflammation account for dysfunctional CD8^+^ T cells.

• ICI is safe in cancer patients who are affected by SARS-CoV-2.

## Introduction

Severe acute respiratory syndrome coronavirus 2 (SARS-CoV-2)-induced diseases are a major concern for global public health in the current years. There are three (1) or four (2) clinical stages for SARS-CoV-2-induced diseases. The four-stage disease includes asymptomatic, mild-to-moderate, severe-to-critical, and chronic-fibrotic states (2). Cancer patients are at a high-risk group (3) in which SARS-CoV-2 is more aggressive in patients with active cancer (4, 5), and the rate of mortality is high in such cases (6). Lung cancer patients affected from SARS-CoV-2 represent more severe conditions. The need for hospitalization is reported in over half of such cases, and death occurs in about a quarter of patients (7). This is mostly due to the immunosuppressive cellular state in the immune ecosystem of cancer (3).

Responses from adaptive immunity emerged in about the first 7 to 10 days of SARS-CoV-2 infection (8). CD8^+^ T cells are the critical cells of adaptive immunity (9). The cells are known as active responders to viral antigens (10). The cells take central roles for controlling viral infection (9) and directly participate in viral clearance (11). CD8^+^ T cells are also considered as the front-line defense against cancer (12) and form a backbone for successful cancer immunotherapy. The irreversible dysfunctional state in CD8^+^ T cells and the scarcity of antigens in antigen presentation machinery are all responsible for the lack of response from solid tumors to immunotherapy (13). Low infiltration or retarded functionality of CD8^+^ T cells will define immune escape and cold immunity (12), such as for tumors like prostatic, pancreatic, breast, ovarian, and colon cancers (14, 15). CD8^+^ T cells overexpressing co-inhibitory receptors on their surface display deviated activation and functionality (16). In adoptive cell therapy (ACT) of cancer, the functionality and specificity of CD8^+^ T cells are enhanced using genetically modified receptors, so-called chimeric antigen receptors. Revitalization of dysfunctional CD8^+^ T cells is also the current focus in cancer immune checkpoint inhibitor (ICI) therapy (13).

CD8^+^ T cells evolve in different processes from life to death including clonal expansion, memory T-cell generation, and exhaustion. Long-lived effector T cells when exposed to a certain antigen undergo a resting state through forming memory cells. Such memory T cells when exposed to the same pathogen will recover their effector activity and render faster responses, thereby providing sustained immunity (17). Patients with asymptomatic or mild SARS-CoV-2-induced disease show strong virus-specific and sustained responses from CD8^+^ T cells (18). SARS-CoV-2-specific CD8^+^ T cells in mild cases show a higher production of interferon (IFN)-γ compared with severe/critical cases (19). By contrast, patients with moderate or severe symptomatic disease represent minimal induction of CD8^+^ T cells (18). The fraction of CD8^+^ T cells is also reduced in patients with a severe (compared with mild) disease (9, 20). These are indicative of the negative impact of SARS-CoV-2-induced diseases on CD8^+^ T-cell fraction and functionality. This review aims at discussing the CD8^+^ T cellular state in SARS-CoV-2-induced diseases and in cancer, focusing on interrelated events and introducing potential management strategies.

## Lymphopenia and Hyperinflammation in SARS-CoV-2-Induced Diseases and the Activity of CD8^+^ T Cells

Lymphopenia or lymphocytopenia is a common characteristic of severe SARS-CoV-2-induced diseases (21, 22). As reported by Wang and colleagues, 69.5% (237/344) of patients in the intensive care unit (ICU) showed lymphocytopenia, which was prominent in non-survivors (23). Lymphopenia occurs due to acute viral infections, which increases the risk of infection-associated hospitalization and mortality. Consistent reduction of T cells is found in peripheral blood of cases with a SARS-CoV-2-induced disease (24). Besides CD8^+^ T cells (9), patients with a severe disease display considerable reduction in the number of CD4^+^ T, NK, and NKT cells. Such reduced presentation is more predominant for CD8^+^ T cells compared with that for CD4^+^ T cells in which the CD4^+^/CD8^+^ T cell ratio is considerably higher in severe (vs. mild) cases (25). Reduction of the lymphocyte count in peripheral blood is presumably indicative of higher inflammatory infiltration into the lung area (24). Patients with a critical disease who have a lymphocyte count lower than 5% following disease onset need intensive care therapy, whereas in cases with a moderate disease variation of such parameter after disease onset is very scare (4). The cell count and characterization of different subpopulations of T cells are evaluated in patients with a SARS-CoV-2-induced disease. A lower absolute number of both CD4^+^ and CD8^+^ T cells in such cases were found (26). The respective counts of CD4^+^ and CD8^+^ T cells negatively associated with patients’ survival are 400 and 300 cells per µL, which were restored after disease resolution (27). Lower rates of proliferation of terminally differentiated T cells in patients with SARS-CoV-2 infection compared with that for healthy subjects were also attested (26). A point here is that patients with active SARS-CoV-2 show a lower absolute number of CD8^+^ T cells. The existing cells are in the hyperactive state (28) and are more prone to turn into an exhaustive state. A low fraction of CD8^+^ T cells is attributed to the development of acute respiratory distress syndrome (ARDS) in patients with active SARS-CoV-2 (29). Lung overload with such hyperactive cells results in the release of high concentrations of chemokines and cytokines, which is a common event in patients with a severe disease (22). This is indicative of the importance of the adequate number of CD8^+^ T cells for providing protection against the virus in which even in the presence of functional CD8^+^ T cells such patients will show a progress in the disease toward ARDS.

Hyperinflammation is a common symptom and a cause of lymphopenia in patients with a SARS-CoV-2-induced disease (24). Hyperinflammation is potentially destructive to host cells. Cytokine surge within the body causes multiorgan injury (22). Patients with a severe disease show a significant increase in the serum levels of IL-6, IL-10, and TNF-α (25). Here, the number of T cells is inversely correlated with serum concentrations of IL-6, IL-10, and TNF-α. The fraction of these inflammatory cytokines was reduced after disease resolution (27). Cases with active SARS-CoV-2 infection show signs of immune activity within the respiratory system (30). It seems that a higher inflammatory infiltration toward the lungs is partially responsible for a decrease in the number of lymphocytes within peripheral blood (24). Here, a moderate disease is correlated with a high T-cell fraction, whereas severe cases show a high fraction of inflammatory monocytes as well as neutrophils (30). Single immune cell landscape was recently characterized in bronchoalveolar lavage fluid of patients displaying varying degrees of SARS-CoV-2-induced disease. Liao and colleagues in this study attested the abundant presence of pro-inflammatory monocyte-derived macrophages in the lavage fluid of cases with a severe disease, while highly clonally expanded CD8^+^ T cells were more observable in the cases with a moderate disease. The amplification index for CD8^+^ T cells for moderate cases was >5 cells compared to the severe/critical cases. In fact, lung macrophages in severe cases may be responsible for local inflammation through promoting the recruitment of inflammatory monocytes and neutrophils, which is mediated *via* CCR1 and CXCR2. By contrast, macrophages in moderate cases produce chemokines for more attraction of T cells, which is mediated through engagement of CXCR3 and CXCR6 (31). Therefore, the significantly higher neutrophil count (vs. lymphocyte count) in severe vs. moderate cases of SARS-CoV-2-induced disease could be asserted, as illustrated elsewhere (9), rendering a high neutrophil-to-lymphocyte ratio (NLR) in such cases (32). The predictive value of NLR in a more severe disease is a focus of a recent systematic review in the area (33). NLR is a marker of systemic inflammation (30) and a reliable and easily measurable parameter in patients with a SARS-CoV-2-induced disease, the measurement of which will help early diagnosis and timely management of this devastating disease (33, 34). Aberrant neutrophil responses occur as a result of exacerbated inflammation, tissue hypoxia, and uncontained viral replication (35). Hypoxia promotes a vicious cycling of neutrophil reactivity in injured lung tissue. In patients with a severe SARS-CoV-2-induced disease, reduced saturation of O_2_ within the blood may cause hypoxia-inducible factor (HIF)-1α activation and excessive neutrophil function in such cases. Neutrophils produce LCN2, HGF, and RETN that their evaluation within plasma is considered as a predictive marker of a critical disease and mortality. A point to consider here is that neutrophil changes are attributed not only to its counts but also to its altered phenotypical states in which patients with a severe (vs. mild) disease show a high fraction of pre-mature or immature cells (36). This is indicative of the impact of SARS-CoV-2 in triggering emergency granulopoiesis and the pre-mature mobilization of bone marrow-derived neutrophil precursors (37). Precise delineation of different subsets of neutrophils can, thus, be considered as a marker of severe SARS-CoV-2 induced disease (38).

### Key Notes

Lymphopenia is a marker of more severe SARS-CoV-2-induced diseases. Extreme T-cell activity within the lungs causes hyperinflammation. NLR measurement is of prognostic value in patients with active SARS-CoV-2.

## CD8^+^ T Cells in Cancer

Signaling pathways and biological mechanisms rendered by CD8^+^ T cells undergo changes in a progressive cancer (39). Reactivation of these cells is important therapeutically even in patients with advanced cancers. Patients with head and neck squamous cell carcinoma (HNSCC), for instance, show higher overall survival (OS) when the fraction of CD8^+^ T cells is increased at recurrence (39).

### Tumoral Infiltration of CD8^+^ T Cells

Efficacy of immunotherapy relies partly on CD8^+^ T-cell trafficking into the tumor area (40). Infiltration of CD8^+^ T cells is low in advanced cold cancers, which is a reason for low or no immunotherapy response from such type of cancer (12). Colorectal cancer (CRC), for instance, shows lower CD8^+^ T-cell density in advanced vs. early stages (40). In patients with advanced pancreatic cancer, low infiltration of CD8^+^ T cells and high expression of programmed death ligand-1 (PD-L1) are associated with high number of cancer stem cells (CSCs), which show immunosuppressive effects (41). In addition, presence of CD8^+^ T cells is also considered as a prognostic marker in breast cancer (42), which is associated with reduced risk of death from the disease (43). By contrast, T-cell infiltration is higher in hot cancers, thereby representing better response to ICI therapy (40). The fraction of cytotoxic T cells (CTLs) is different in three categories of tumor patients. Based on outcomes of a quite recent study, patients in the “inflamed” group represented intraepithelial CTLs of >500 cells/mm^2^, whereas cases in the “immune desert” group had a stromal CTL fraction of ≤50 cells/mm^2^. Patients who are not included in this cutoff range were placed in the category of the “immune excluded” group, showing a quite low number of intraepithelial CTLs (44).

#### Key Note

Patients with diverse immune landscapes display different fractions of CD8^+^ T cells.

### Cross Talks Between CD8^+^ T and Other Cells of the Tumor Microenvironment

Cancer-associated fibroblasts (CAFs) are the dominant cells of the tumor microenvironment (TME) that take multifaceted roles for promoting tumor aggression and metastasis (45). CAFs suppress the intra-tumoral migration of CD8^+^ T cells (46) and exclude their contact with cancer cells. This is mediated through release of C–X–C chemokine ligand 12 (CXCL12), as well as promoting extracellular matrix (ECM) stiffness (45). High activity of CAFs within the edge or invasive front of cancer and their exposure to the hypoxic conditions in this tumor region is a reason for the cold immunity of this area. CD8^+^ T cells are immunologically ignored in the edge area. The cells are induced by macrophage type 1 (M1) cells to recruit into the core area of tumor, rendering higher sensitivity of this tumor region to therapy (47). M1 cells are called classically activated cells. The cells represent a pro-inflammatory signature and have antitumor activities (48). CD8^+^ T cells possibly have a negative cross talk with myeloid-derived suppressor cells (MDSCs) in which a fall in the number of MDSCs infers a rise in the fraction of CD8^+^ T cells (49).

**Figure 1:**
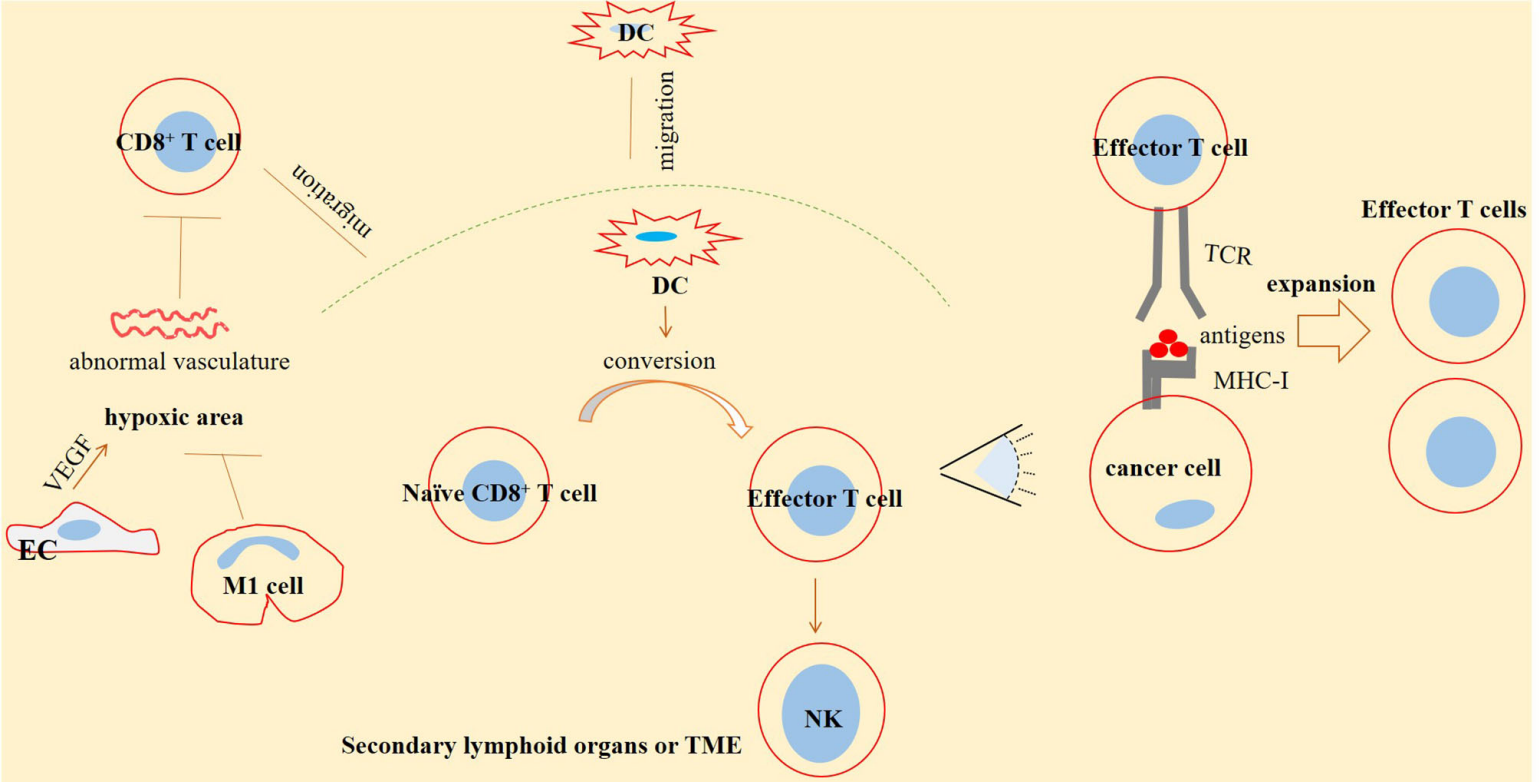
CD8^+^ T-cell infiltration and effector function against cancer. CD8^+^ T cells are infiltrated into the tumor microenvironment (TME) and secondary lymphoid organs. Here, CD8^+^ T cells are converted into effector T cells under exposure to dendritic cells (DCs) migrated toward the area and form a functional circuity with DCs and natural killer (NK) cells. A normal vasculature is an inducer of CD8^+^ T-cell migration toward the tumor area. Generally, tumor vasculature is abnormal in solid cancers, especially in advanced stages, which is a reason for higher immune escape and resistance. High vascular endothelial growth factor (VEGF) release from endothelial cells (ECs) is a key promoter of such abnormality. High VEGF is linked with hypoxia and a leaky vasculature. Macrophage type 1 (M1) cells are antitumor cells of TME that are acting for strengthening immune effector function through promoting vascular normality. T-cell receptors (TCRs) expressed on infiltrated CD8^+^ T cells are able to detect antigens bounded to the major histocompatibility complex-1 (MHC-1), expressed on the surface of tumor cells, a result of which is the expansion of effector T cells able to act against cancer.

CD8^+^ T cells, dendritic cells (DCs), and natural killer (NK) cells form a net of functional circuity. The activity of NK cells is induced by CD8^+^ T cells. Such functional circuity will lead to a co-operative activity, maximizing effector functions of these critical cells against cancer (50). CD8^+^ T cells form CTLs upon exposure to the antigen-presenting cells (APCs), such as DCs. CTLs recognize major histocompatibility complex-1 (MHC-1)-bounded antigens expressed on target cells (17). Naïve CD8^+^ T cells will become activated when they are under exposure to the mature DCs (51) (**Figure 1**).

#### Key Note

Co-operative activity within the NK/DC/CD8^+^ T-cell axis is effective for retarding immunological ignorance.

#### Inverse Cross Talking Between CD8^+^ T Cells With Regulatory T Cells in SARS-CoV-2-Induced Disease and Cancer

Regulatory T cells (Tregs) are the key components of adaptive immunity; their activity is attributed to the maintenance of tolerance to self-antigens, thereby prohibiting autoimmune diseases (52). In the context of cancer, Treg depletion increases CD8^+^ T-cell responses, which is mediated partly *via* recovering the functionality of exhausted CD8^+^ T cells (53). Tregs prevent cytokine storm and reduction of tissue damage (24). Excessive inflammation is a hallmark of SARS-CoV-2-induced diseases, and Tregs are for restraining ample inflammatory events (54). To support this idea, high Treg number is detected in patients with milder symptoms from SARS-CoV-2-induced diseases (2). Patients with severe disease experience a low number of Tregs (32). A question here is whether Treg-depleting strategies are applicable for cancer patients with a severe SARS-CoV-2-induced disease. T cells show higher responses to self-antigens when the number of FOXP3^+^ Tregs is reduced. This will lead to the development of autoimmune-like responses from T cells to self-antigens, a result of which is the depletion of immunological resources (55). Thus, Tregs act as a double-edged sword through suppressing excessive inflammation, while possibly lowering host defense to act against SARS-CoV-2-related infection (28). In fact, Tregs are possibly attributed to the reduction of antiviral defense in patients with the early-stage SARS-CoV-2-induced disease, while they ameliorate inflammation-induced organ damage in cases with the late-stage disease (28). Assessment of baseline Treg levels after hospital admission for SARS-CoV-2-induced diseases is important due to predicting clinical worsening of the disease. Based on the outcomes of a study, patients harboring reduced baseline levels of Th1, Th2, and Tregs are more prone to display signs of clinical deterioration (56).

**Figure 2:**
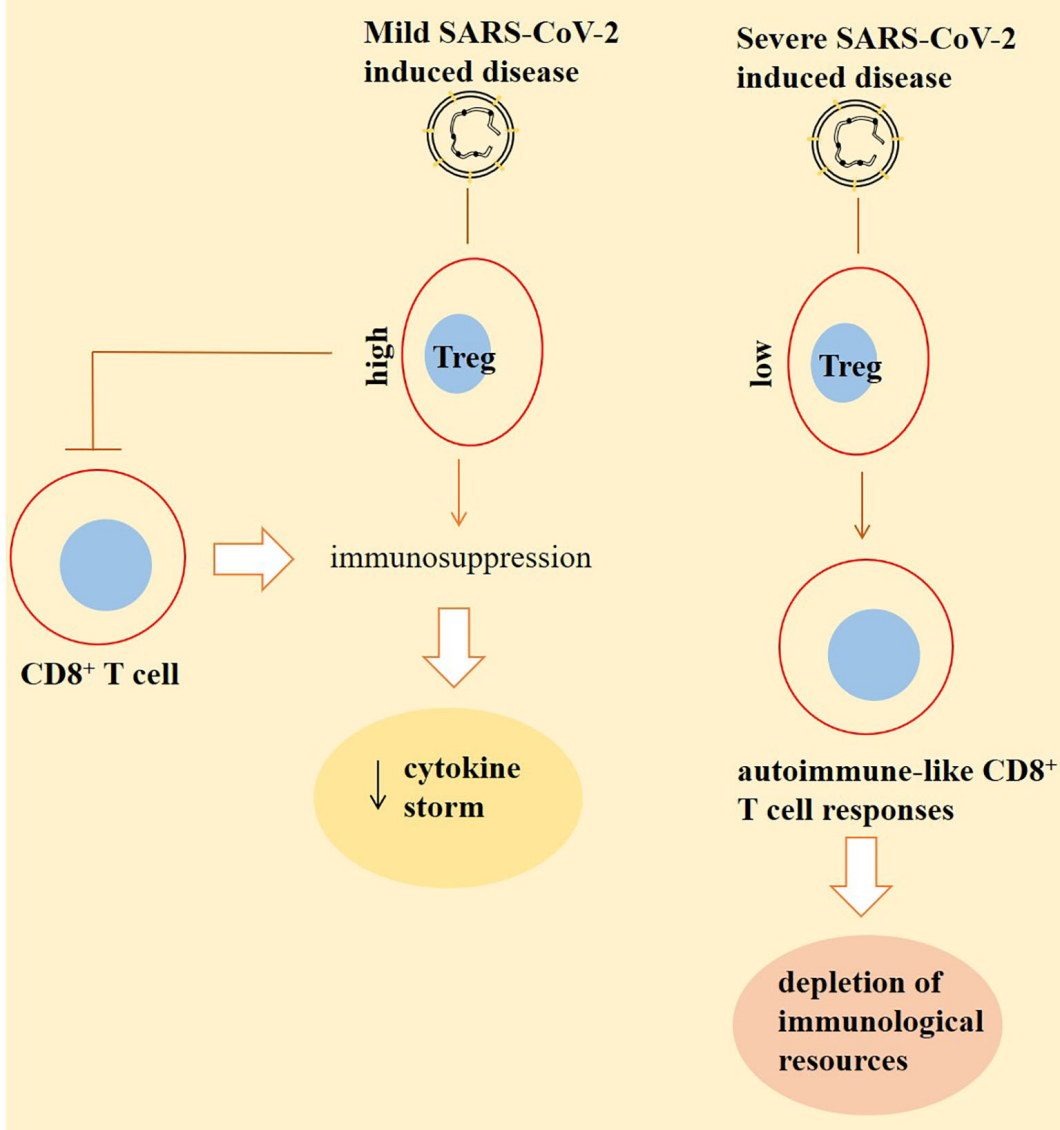
Inverse cross talking between CD8^+^ T cells with regulatory T cells (Tregs) in patients with a SARS-CoV-2-induced disease. Tregs are high in patients with a mild disease. This will result in immunosuppression partly by suppressing the activity of CD8^+^ T cells. Such immunosuppression further reduces the extent of cytokine storm, thereby lessening the severity of the condition. By contrast, the number of Tregs is low in severe cases. This will cause responses from CD8^+^ T cells to self-antigens, and the cells are representing autoimmune-like states, which results in the depletion of immunological resources to combat against the virus. The outcome of this autoimmunity is higher viral persistence and a more severe condition.

The Treg compartment displays unique changes in patients with a severe SARS-CoV-2-induced disease (57). Such cases show divergent deviations and perturbations in Treg phenotypes (54). Based on outcomes of one study, the fraction of Tregs is reduced in hospitalized patients, whereas cytotoxic T helper and follicular helper T cells show an increased fraction (58). Galván-Peña and colleagues reported that patients with a severe disease had a distinct transcriptomic profiling, delineated by increased intracellular level of the transcription factor FOXP3, which is associated with poor outcomes. Such an increase in the FOXP3 expression was in coincidence with a rise in the percentage of Tregs in most of the patients (but not all). Interestingly, in patients with a rise in FOXP3 and Tregs there was a reduction in the expression of the transcription factor CD45RA, which is a marker of naïve Tregs. However, Tbet, which controls preferentially responses from Th1 cells, was overrepresented on Tregs in patients with a severe disease. It seems that blood Tregs from patients with a SARS-CoV-2-induced disease turned into the cells resembling that of tumor-infiltrating Tregs (54). The level of naïve Tregs is increased significantly postinfection. Due to the dual activity of Tregs in suppression of immune responses and promotion of tissue repair, an expansion in the level of naïve Tregs can be an effort for restoring equilibrium in the Treg pool upon facing both tissue injury and inflammation (59). Treg depletion is a possible strategy for enhancing CD8^+^ T-cell functionality in cancer patients, but as is mentioned above, it may strengthen viral persistence when such cases also have SARS-CoV-2-related infection (**Figure 2**).

#### Key Note

Treg depletion may strengthen viral persistence in cancer patients with active SARS-CoV-2. Divergent deviations of Tregs exist among patients with a severe SARS-CoV-2-induced disease, which is seemingly resembling of Tregs in cancer patients.

## CD8^+^ T-Cell Expression Profile in SARS-CoV-2-Induced Disease and Cancer

**Figure 3:**
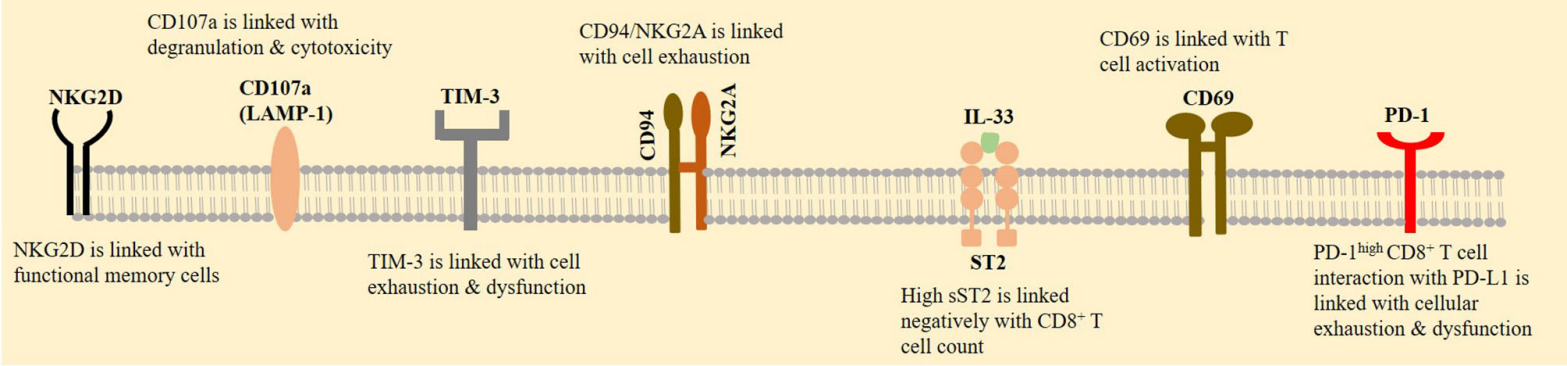
CD8^+^ T-cell expression profile. A rise in the expression of natural killer group 2, member A (NKG2A) is contributed to the exhaustion of CD8^+^ T cells in patients with a SARS-CoV-2-induced disease, whereas natural killer group 2, member D (NKG2D) is contributed to the functional memory T cells. CD8^+^ T-cell activation is also promoted by overexpression of CD69. CD107a is another marker of cellular activation. By contrast, T-cell immunoglobulin mucin-3 (TIM-3) and programmed death 1 receptor (PD-1) are checkpoints that their increased expression on CD8^+^ T cells is indicative of their impaired functionality. Interleukin 33 (IL-33) activity enhances CD8^+^ T-cell functionality, proliferation, and infiltration. Soluble suppressor of tumorigenicity 2 (sST2) is a receptor that binds with IL-33 and is contributed to the local limitation of off-target IL-33 activity.

NK group 2 member A (NKG2A) is a receptor that belongs to the lectin family and forms a heterodimer with its co-receptor CD94 (also called KLRD1). CD94/NKG2A sends inhibitory signals (60), and its enhanced expression is seen on exhausted NK and CD8^+^ T cells in patients with a SARS-CoV-2-induced disease. The concomitant reduced expression of CD94/NKG2A occurs in convalescent individuals (4). The expression of NKG2A on CD8^+^ T cells is high in patients with an acute severe/critical disease (61), delineating the activity of this inhibitory receptor in promoting the severity of condition in patients with a SARS-CoV-2-induced disease. CD94/NKG2A surface co-expression is reported in about 50% of CD8^+^ T cells, and its selective expression is linked with poor clinical outcomes in HNSCC patients. In fact, the induction of NKG2A on CD8^+^ T cells infiltrated into the tumor area is a mechanism of immunosuppression in patients with cancer. Blockade of this inhibitory receptor can improve the efficacy of vaccination therapy against cancer, represented by delayed tumor recurrence after therapy (62). By contrast, natural killer group 2, member D (NKG2D, also called CD314), is an activating receptor of the lectin family. NKG2D is expressed on the surface of NK and CD8^+^ T cells and acts as a killer of infected or cancer cells (63). The constitutive expression of NKG2D on human CD8^+^ T cells (64) is linked positively with their functional memory features (65). The activity of NKG2D on CD8^+^ T cells needs the parallel activation of the T-cell receptor (TCR). This is different from the active NKG2D on NK cells in which the killing responses to NKG2D are unleashed without a need for concurrent TCR activity (64). The expression of CD107a is a marker of cellular activation that is linked with cytotoxic degranulation (66). CD8^+^ T cells from patients with a SARS-CoV-2-induced disease also overexpress CD69 and T-cell immunoglobulin mucin-3 (TIM-3) (67). CD69 is a marker of T-cell activation, and TIM-3 is the checkpoint and co-inhibitory molecule (**Figure 3**).

Interleukin 33 (IL-33) increases CD8^+^ T-cell functionality (68) and is contributed to its proliferation and infiltration of CD8^+^ T cells, thereby acting as a suppressor of tumor growth (69). The soluble suppressor of tumorigenicity 2 (sST2, also called IL-1RL1) is a receptor that binds with IL-33 and is contributed to the local limitation of off-target IL-33 activity and thereby prohibits inadequate inflammatory responses (68). sST2 is considered as a prognostic biomarker in SARS-CoV-2-induced diseases (70). There is a high serum level of sST2 and a negative correlation between serum sST2 with CD8^+^ T cell count in patients with an active SARS-CoV-2 infection (71). Analysis of bronchoalveolar lavage fluid in patients with mild-to-severe SARS-CoV-2-induced diseases shows a population of cells with the activity of IL-33 production and an increase in their fraction upon disease progression (72). The activity of IL-33 contributed to tissue remodeling, homeostasis, and repair during acute SARS-CoV-2 infection, mediated partly *via* increasing the number of CD8^+^ T cells. By contrast, during chronic SARS-CoV-2 infection, the regulatory impact of sST2 over IL-33 activity is overwhelmed, thereby causing an over-release of IL-33 and persistent inflammatory response from CD8^+^ T cells, which finally causes tissue damage (68). During severe infection with SARS-CoV-2, tight interactions between active immune cells with the airway epithelium will cause epithelial damage, a result of which is high IL-33 release. Over-release of IL-33 impairs antiviral activity of CD8^+^ T cells and initiates inflammatory responses within the lungs. A consequence of such dampened antiviral immunity is hyperinflammation, delayed viral clearance, and more severe infections (73) (**Figure 1**).

### Key Note

High expressions of NKG2A and NKG2D are representative of the respective CD8^+^ T-cell exhaustion and functional memory features. IL-33 is a tumor suppressor, but its upregulation in lung epithelial cells is contributed to the hyperinflammation in patients with severe SARS-CoV-2.

## CD8^+^ T Cellular States in SARS-CoV-2-Induced Disease and Cancer

### CD8^+^ Memory and Effector T Cells

Successful immunotherapy relies on recovering antigen-specific effector memory T cells within the TME (74). Memory CD8^+^ T cells are important for promoting sustained immunity against cancer (75). Central memory, effector memory, resident memory, and stem-like memory cells are different subsets in this category. Resident memory T cells are placed in local tissue areas and contribute to the immediate response to secondary infection, whereas central memory and effector memory T cells circulate within blood, and their target organs are secondary lymphoid tissues (76).

**Figure 4:**
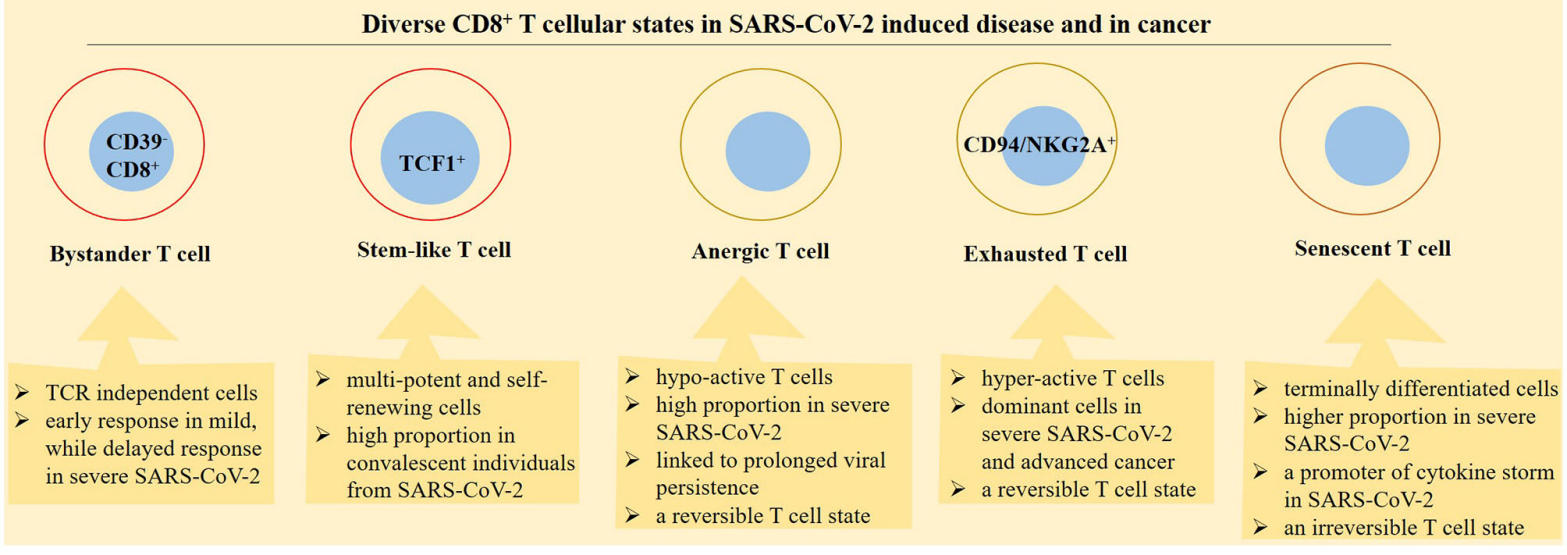
Diverse functional profile of different CD8^+^ T-cell states in patients with a SARS-CoV-2-induced disease and cancer. Bystander, stem-like, anergic, exhausted, and senescence are diverse cellular states in relation with CD8^+^ T cells. The activity of bystander cells is independent on T-cell receptors (TCRs). Stem-like CD8^+^ T cells are placed in designated immune niches and act for formation of effector T cells. Anergic T cells are hypo-reactive, whereas exhausted T cells are hyperreactive but both cellular states are reversible. Senescence is a term used for terminally differentiated T cells and is considered as an irreversible T-cell state.

**Figure 5:**
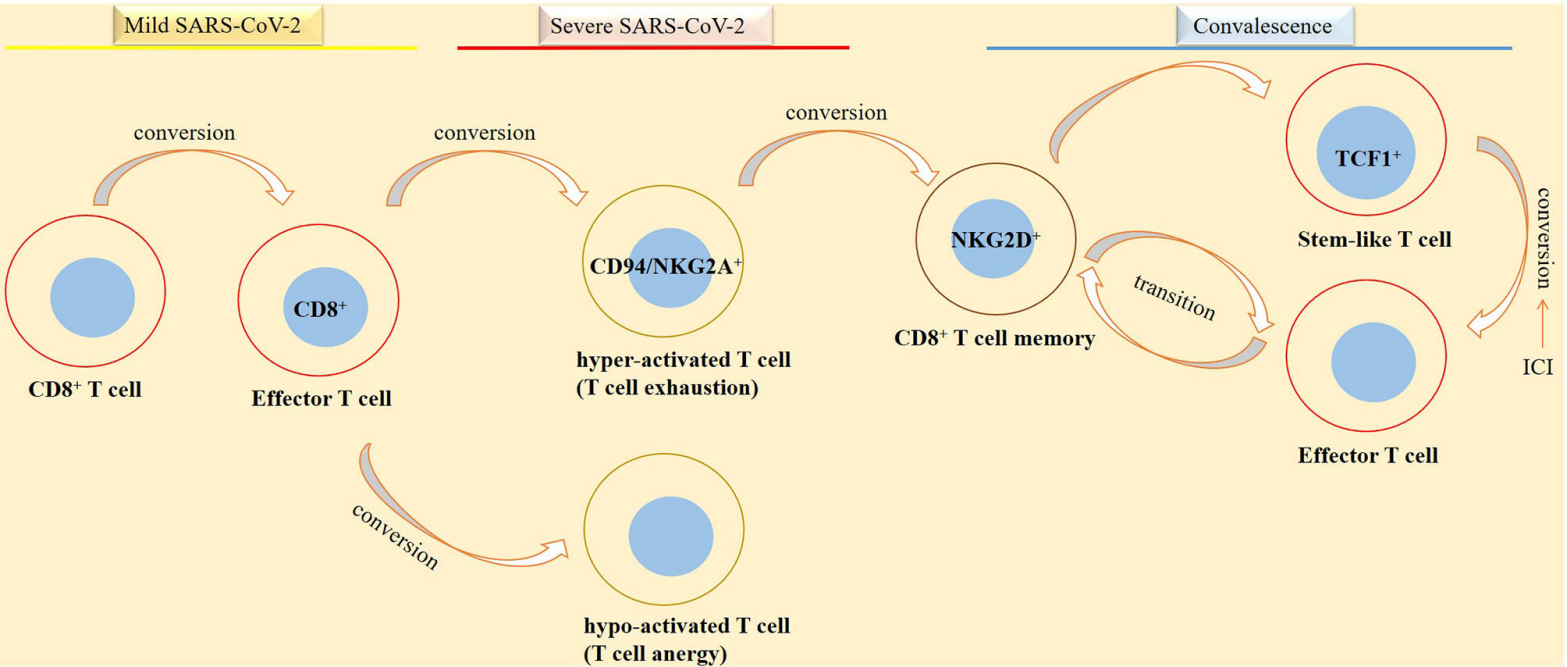
Phases of SARS-CoV-2 induced disease in relation with CD8^+^ T-cell activity. CD8^+^ T cells take diverse functionality in different phases of SARS-CoV-2 induced disease. In patients with a mild disease CD8^+^ T cells are converted into functional effector cells for taking action against the virus. By contrast, in patients with a severe disease, CD8^+^ T cells are converted into either a hyperactive or a hypo-active phenotype, designated by the respective exhaustive and anergic cellular state with both representing T-cell dysfunctionality against the virus. In convalescent individuals from a SARS-CoV-2-induced disease, there is a different story. In such cases, stem-like and effector cells are formed in order for promoting action against viral reentry or reducing the hazard effect of the virus on tissues or organs.

Memory T cells are considered as a hallmark of antiviral immunity (77). The cells are presented in convalescent individuals from SARS-CoV-2-induced diseases (78) and are generated as a response to vaccination, infection, or after viral reexposure (8). Memory T cells are important for quick responses against infection, and their presence is a reason for the low severe condition experienced by patients upon viral reentry (21, 79). During recovery from SARS-CoV-2-induced diseases, dysfunctional T cells are converted into functional CD8^+^ memory T cells (61). Bert and colleagues evaluated T-cell responses in patients that recovered from SARS, the disease related to the SARS-CoV infection. They noticed the presence of memory T cells reactive to the SARS-CoV N protein 17 years after the SARS outbreak in 2003 (80). The dynamic transition between effector and memory T cells is also reported in SARS-CoV-2-specific CD8^+^ T cells (81). Such CD8^+^ memory T cells will reach a peak at about 2 weeks after infection with this virus. The cells are also detectable even after 100 days (8), and their response against SARS-CoV-2 is maintained for 10 months. The cells are able to form stem-like memory cells (82), but their fraction is reduced over time (8) (**Figures 4**, **5**).

### Key Note

CD8^+^ memory T cells provide protection against viral reentry, but their fraction is reduced over time.

### Stem-Like CD8^+^ T Cells

T-cell memory in cancer and cancer immunotherapy relies on the formation of antigen-specific stem-like progenitor cells (83). Stem-like T cells are T-cell factor (TCF)-1^high^ memory cells that represent higher proliferative capacity and produce a larger number of effector cells, as compared with other T-cell memory subsets (76). Stem-like memory T cells are multi-potent and have self-renewal potential (84). The activity of stem-like CD8^+^ T cells contributes to the complete regression of tumor and tumor-infiltrating lymphocyte (TIL) persistence in patients receiving ACT (85). Two subtypes of stem-like progenitor memory of CD8^+^ T cells are identified: progenitor cells committed to functional lineage that lack the T-cell immunoreceptor with Ig and ITIM (TIGIT) and programmed death 1 receptor (PD-1), and those committed to dysfunctional lineages that express PD-1 and TIGIT and represent exhausted-like T cells (83). Functional stem-like CD8^+^ T cells are negative for CD39 and CD69 (85) but highly express CD28 and TCF1 and are characterized as TIM-3^-^CD28^+^TCF1^+^ cells (86). Such cells are dependent on TCF1 for recalling their self-renewal potential (87). TCF1^+^PD-1^+^CD8^+^ T cells share features of central memory T cells but do not represent an effector signature (88). In fact, progenitor T cells with TCF1^+^PD-1^+^ signature are referred to as memory-like cells, whereas cells with a TCF1^-^PD-1^+^ expression profile are referred to as exhausted cells (87). The identity of the memory CD8^+^ T-cell pool is important from the therapeutic standpoint. Is this send a message to use ICI therapy for reinstating functional stem-like cells in immune niches? Stem-like TCF1^+^PD-1^+^CD8^+^ T cells are able to form differentiated T cells (TCF1^-^PD-1^+^CD8^+^) as a response to ICI, and their contribution is vital for controlling cancer upon ICI treatment. Therefore, a proliferative response in CD8^+^ T-cell progeny is occurring in patients receiving ICI (89).

Generally, T cells will lose their effector function and promote terminally differentiated exhausted phenotype when they are under exposure to chronic infections or are encountered to the immunosuppressive milieu of TME (88, 90, 91). TCF1^+^PD-1^+^CD8^+^ T cells contribute to sustained immunity in patients with chronic viral infection (88), such as SARS-CoV-2-induced disease (84). Stem-like CD8^+^ memory T cells exist in convalescent SARS-CoV-2 individuals, and their presence is possibly indicative of long-term protection against the virus (79). Based on findings from a study, stem-like memory T cells are successfully generated in convalescent individuals, which reached a peak at about 120 days post-symptom onset (84). This is indicative of the importance of these cells in patient recovery from SARS-CoV-2-induced diseases (**Figures 4**, **5**).

### Key Note

The activity of stem-like CD8^+^ T cells is contributed to the complete regression of tumor and long-term protection against SARS-CoV-2.

### Hyper/Hypo Active and Senescent T Cells

Hyperactivation or exhaustion is a dominate immune response in severe SARS-CoV-2-induced diseases (67). Exhaustion is a term used for progressive weakness in cytokine generation and cytotoxicity. CD8^+^ T cells are turned into an exhausted state upon chronic exposure with cancer antigens, viral infection, or in the context of autoimmune disease. The activity of these cells in chronic inflammatory conditions is inefficient, and they are not able to clear the infection (90). Thus, exhausted T cells are epigenetically distinct from memory and effector T cells through displaying defective functionality. Exhaustion is, in fact, a differentiation process that eventually causes cell death (17). CD8^+^ T cells sustain the state of exhaustion even after resolution of viral infection. Such exhausted cells show lower clonal expansion and lower encoding for molecules related to the cytotoxicity but higher rates of genes responsive to type 1 interferon (92). Kusnadi and colleagues in a study evaluated the nature of virus-reactive CD8^+^ T cells in single-cell transcriptomes of patients with a SARS-CoV-2-induced disease. Exhausted SARS-CoV-2-reactive cells showed higher frequency and represented lower inflammatory and cytotoxicity features in patients with a mild disease. By contrast, in patients with a severe disease SARS-CoV-2-reactive cells in the dominant non-exhausted subtype showed a feature of CD8^+^ T-cell memory cells, represented by enrichment with transcripts related to the antiapoptotic, pro-survival, and co-stimulation. Such cells displayed different characteristics with that for cells reactive to influenza, which is indicative of diverse functional features and transcriptome profiling for CD8^+^ T cells upon exposure to the various types of viruses (93). PD-1^−^CTLA-4^−^TIGIT^−^ CD8^+^ T cells is a non-exhausted subset that shows reduced frequency in severe diseases. Inhibition of PD-1, CTLA-4, and TIGIT is an effective way for maintaining the antiviral activity of CD8^+^ T cells (94).

Anergy is a state of impaired or hypo-reactivity (95) that is occurring in cells under suboptimal stimulation (96). T-cell anergy occurs in tumors (96) and is referred to both CD4^+^ and CD8^+^ T cells (97). Anergy is different from exhaustion and senescence. Anergy occurs during T-cell priming, whereas T-cell exhaustion occurs in cells with prior effector function but becomes silenced gradually due to the continuous presentation of antigens. Senescence is a state of growth arrest in a cell undertaking extensive proliferation due to repeated stimulation (98). Senescent T cells are terminally differentiated and represent telomerase shortening and irreversible (or stable) cell-cycle arrest (96). Thus, although both senescent and exhaustive T cells are under continuous stimulation, the latter cell type can retain its functionality under exposure to appropriate stimuli (99, 100). Exhausted T cells display elevated inhibitory checkpoints, such as PD-1 and TIM-3, which do not occur in senescent T cells. A distinct phenotype is seen in senescent cells in which the cells show downregulation of co-stimulatory molecules including CD27 and CD28 but highly express markers like CD57 (101, 102).

T cells may turn into anergic state upon exposure to an immunosuppressive TME (97). Tumor-specific T cells show dysfunction due to exposure to several inhibitory signals from the complex TME (96). TME is represented by insufficient nutrient provision for immune cells. CD8^+^ T cells take an optimal function when they are under exposure to a normal high-glucose content, whereas the cells upon exposure to a hypoglycemic milieu will transit into energy and finally undergo apoptosis (103). DC immaturity, weak co-stimulation, and/or the activity of inhibitory signals are factors related to the insufficient or suboptimal presentation of tumor antigens on T cells (97). The immunosuppressive TME presents high number and activity of Tregs. Tregs prime DCs to induce anergy in conventional T cells. This is mediated in a CTLA-4-dependent manner in which the constitutive expression of this checkpoint on Tregs results in trans-endocytosis of B7 ligands on DCs. This is followed by degradation of such ligands, which interferes with the CD28 co-stimulatory capacity of DCs (104). Thus, immature DCs in TME are not able to provide optimal T-cell activation due to lacking ligands for engaging with CD28 (105).

T-cell anergy is also considered as a hallmark of SARS-CoV-2-induced disease and is correlated with severity of the condition (95). Results of a study showed more pronounced hyporeactive T cells in hospitalized patients with a SARS-CoV-2-induced disease, and such severe hyporeactive T cells are correlated with prolonged viral persistence. SARS-CoV-2-mediated T cell hypoactivity is independent on the activity of immunosuppressive drugs administered to such patients. Such hyporeactive cells were generated due to extrinsic T-cell factors and were due to plasma components, and they were partially recovered by IL-2 (106). Hopefully, anergic T cells can be reversed after recovery from critically ill SARS-CoV-2 induced diseases.

Exposure to IL-2 partially recovered hyporeactive T cells induced by SARS-CoV-2 (95, 106) and cancer. PD-1 promotes anergy in CD8^+^ T cells through hampering the autocrine production of IL-2 from the cells. IL-2 re-complementation, by contrast, recovers the activity of anergic T cells irrespective of PD-1 expression on the cells (107). IL-2 inhibits anergic T cells through induction of JAK and mTOR and mediates its effects through suppressing the expression of genes related to anergy induction including Cbl-b and Ikaros (108). There are also transcription factors, such as nuclear factor of activated T cells (NFAT) and Egr2/3 related to the clonal T-cell anergy (109). NFAT is an important regulator of T-cell activation; its activity is related to the induction of a hyporesponsive cellular state (exhaustion or anergy) in both CD4^+^ and CD8^+^ T cells (110). Egr2 induces Ndrg1, which is kept at high levels in anergic T cells at resting state. Ndrg1 is, thus, a factor related to the clonal T cell anergy where its degradation is induced by IL-2 treatment (109).

T-cell senescence occurs in patients with chronic viral infections, autoimmune disorders, and cancer (101, 111). Malignant tumors induce T-cell senescence in order to evade from immune surveillance (101). Senescent T cells are also contributed to the pathogenesis of SARS-CoV-2-induced diseases (112). Severe infection with SARS-CoV-2 mimics a senescence immune state. The proportion of senescent CD8^+^ T cells is higher in patients with severe (vs. mild) disease. Such senescent cells are not able to produce cytotoxic molecules. Instead, they are equipped with senescence-associated secretory phenotype (SASP). SASP is a term used for a broad spectrum of molecules secreted by senescent cells, which might be a promoter of cytokine storm in patients with severe SARS-CoV-2 induced disease (111). Immunosenescence augments susceptibility to SARS-CoV-2 and blunts the efficacy of vaccination. Senescent immune cells are higher in aged individuals, which is related to the retarded immunity to vaccination therapy among elderly populations (113). Younger cases with chronic viral infection also show senescent CD8^+^ T cells (102) (**Figure 5**).

### Key Note

T-cell exhaustion, anergy, and senescence are occurring in severe SARS-CoV-2-induced diseases and cancer.

### Bystander CD8^+^ T Cells

Bystander T cells are not pathogen specific, but they are able to impact the course of immune responses (114). The cells are CD39^-^ CD8^+^ (115), and their activation is independent on TCR signaling, rather than occurring in the context of autoimmune diseases, infection, and cancer. Such TCR-independent T-cell activation is seen mainly in CD8^+^ T cells (65). The quality of T cells infiltrated into different types of human cancers have recently been described. Bystander CD8^+^ T cells show high fractions in patients with non-melanoma cancers, such as ovarian and renal cancers (116). Bystander-activated CD8^+^ T cells release cytokines, such as IFN-γ that support immune defenses against infection. Cytokine release from activated bystander cells occurs even when the cells are not stimulated by antigens. Thus, due to not being specific, the cytolytic activity of these cells may cause host tissue damage (114, 117). For instance, in non-small cell lung cancer (NSCLC) patients under treatment with immunotherapy, a high fraction of bystander cells within the lungs can impair immune responses and enhance the rate of toxicities (118). Early responses from bystander CD8^+^ T cells are seen in cases with mild or asymptomatic SARS-CoV-2-induced disease. Such cases show no signs of systemic inflammation. This is comparable with delayed bystander CD8^+^ T-cell responses and vigorous systemic inflammation in patients with severe disease (119).

### Key Note

Early response from bystander CD8^+^ T cells is protective against SARS-CoV-2-induced diseases, while their high fractions impair immune responses in cancer patients.

## Antiviral vs. Antitumor CD8^+^ T Cells

Antiviral CD8^+^ T cells often represent higher polyclonality, frequency, and functionality compared with antitumor CD8^+^ T cells. Suggested reasons for such higher immunological competence in antiviral CD8^+^ T cells are as follows: (1) antiviral CD8^+^ T cells are generated as a response to highly immunogenic viral antigens (120), and (2) antitumor CD8^+^ T cells represent diminished activity due to being under exposure to the immunosuppressive TME (120, 121). To explain the latter, solid cancers develop a microenvironment that includes complex signaling along with several cells. Both antitumor and pro-tumor cells existed in this ecosystem, but a tumor with a progressive stage and the number of antitumor cells far exceed that for pro-tumor cells. This occurs due to higher infiltration of pro-tumor cells or even from altered functionality of antitumor cells. The heterogeneity among cells within the TME along with complex conditions including nutrient deprivation, hypoxia, chronic inflammation, and altered redox systems all indicate constant and more pronounced impact over antitumor cells of the immune system, CD8^+^ T cells in particular. Thus, it is fair to assert that the quality of CD8^+^ T cells in an aggressive tumor that is developed toward metastasis is far diminished or may be taken by a terminally exhausted state, which may not be recovered after therapy.

### Key Note

Immunological competence is higher in antiviral CD8^+^ T cells compared with antitumoral CD8^+^ T cells.

## CD8^+^ T Cells in Convalescent Individuals From SARS-CoV-2-Induced Diseases

Rapid and robust responses from CD8^+^ T cells along with responses from Th1 cells efficiently curtail viral replication and viral-related antigen production (18). Specific CD8^+^ T-cell responses are identified in post-symptomatic individuals who had a persistent SARS-CoV-2-induced disease (based on PCR data) and that the persisted disease was not contagious in such individuals (24). Based on one study, CD8^+^ T cells are detected in 70% of convalescent cases (122), and in another work SARS-CoV-2-specific CD8^+^ T cells are detected in 87% of convalescent patients. This is comparable with the 53% rate of SARS-CoV-2-specific CD8^+^ T cells in the acute phase of the disease (123). T cells display active cytotoxic phenotype during the acute phase of the disease (79). The cells are IFN-γ positive in majority of samples from patients with an acute disease or in convalescent individuals (123). CD8^+^ T cells from convalescent individuals have broad activities through targeting the whole viral proteome and both structural and non-structural epitopes (81). SARS-CoV-2-specific CD8^+^ T cells are detectable for 5 months after convalescence from the severe/critical disease (61). However, transfusion of convalescent plasma from recovered individuals is not effective for recovering CD8^+^ T-cell effector function in patients with a severe disease in which the fraction of active CD8^+^ T cells is reduced at 28 days’ post-transfusion (124).

### Key Notes

Reduction of active CD8^+^ T cells shortly after convalescent plasma transfusion from patients with severe SARS-CoV-2 induced disease is indicative of the weakness of this approach for proving protection on other individuals.

## CD8^+^ T-Cell Responses in Different Types of Vaccination Against SARS-CoV-2

CD8^+^ T-cell responses are elicited by several vaccines against SARS-CoV-2 (125). BBV152 (also called Covaxin) is an inactivated SARS-CoV-2 vaccine formulated by a collaborative work between Bharat Biotech and the Indian Council of Medical Research. Administration of BBV152 to healthy adults enhanced CD8^+^ T-cell responses (126). Responses from these cells are expected to facilitate patient recovery and possibly prevent severe diseases. In regard with the Ad26.COV2.S vaccine developed by Johnson & Johnson company, CD8^+^ T-cell responses were seen in the respective 51% and 64% of cases receiving low and high doses of vaccination and that the rate was considerably lower in older individuals. Response from CD8^+^ T cells to the mRNA vaccine developed by Pfizer-BioNTech was 76% (127). The AZD1222 (AstraZeneca) vaccine is developed based on the S protein-encoded replication-deficient chimpanzee adenoviral vector (ChAdOx1) (128). Responses from CD4^+^ Th1 and CD8^+^ T cells were characterized after AZD1222 vaccination. Two doses of AZD1222 showed considerable responses from both cell types in all adult age groups (18–85 years old). In addition, both cell types showed a high rate of poly-functionality, which is representative of the strengthening of the power of the immune system to act against SARS-CoV-2 spike protein (129).

BNT162b2 is a nanoparticle-formulated RNA vaccine developed by Pfizer and encodes the full-length spike protein of SARS-CoV-2. Based on outcomes of one study, the efficacy of BNT162b2 for preventing SARS-CoV-2 was 95% (130). BNT162b2 vaccination showed S-specific CD8^+^ T-cell expansion in most participants. A high fraction of CD8^+^ T cells was able to produce IFN-γ and that the cells had effector-memory phenotype. In addition to this, Th1 CD4^+^ T cells also showed expansion in most of the participants. These are indicative of the BNT162b2-mediated combined cellular and humoral responses from the immune system for providing protection against SARS-CoV-2-induced diseases (131). In a study, T-cell responses were measured in healthcare workers previously infected with SARS-CoV-2 and received one dose of BNT162b2 vaccination and in individuals without prior infection with such virus but receiving one or two doses of the vaccine. Results showed higher T-cell responses against spike proteins in previously infected cases than infection-naïve individuals. T-cell responses in previously infected cases receiving one dose of vaccine were equivalent with those for infection-naïve individuals receiving two vaccine doses (132). This has also been approved by another study in which prior SARS-CoV-2 infection resulted in more responses from B and T cells to the vaccination with BNT162b2 and that the high antibody concentration in individuals with previous exposure to the virus and receiving one dose of vaccine was comparable with cases receiving two doses of vaccine. This is indicative of the adequate protection evolved by the one-dose vaccine for boosting immunity against the severe disease in recipients with prior exposure to SARS-CoV-2 (133). From what is discussed above, it could be asserted that T cells provide a level of immunity in cases with prior exposure to SARS-CoV-2, and their contribution is seemingly important for reducing the possibility of progressive disease in cases with second exposure to the virus. In fact, the activity of T cells will strengthen the efficacy of vaccination therapy against the virus. Outcomes of a study on gynecologic malignancies showed that cancer patients recovered from SARS-CoV-2 infection may also be protected from reinfection by such virus quite similar to that in non-cancer SARS-CoV-2 cases (134). This presumably indicates that T cells recalled for providing protection against SARS-CoV-2 are specific to that virus and being different from what acted against cancer. However, this is not indicative of insufficiency of cancer-specific CD8^+^ T cells on SARS-CoV-2. Potentiating the fraction and activity of such cells in cancer patients can also be effective to provide protection against SARS-CoV-2, as discussed further.

### Key Note

SARS-CoV-2 vaccines act for recovering CD8^+^ T-cell responses.

## CD8^+^ T Cells and Checkpoints in Cancer Patients With SARS-CoV-2

Immune checkpoints are referred to as molecules that act as gatekeepers in immune responses and are considered as immune regulators. Checkpoints used for ICI therapy are TIM-3, cytotoxic T lymphocyte associated antigen-4 (CTLA-4), and PD-L1 (99, 100). In addition, TIGIT is a novel checkpoint receptor identified on the surface of CD8^+^ T cells, and its expression is related to the T-cell exhaustion and immune escape in patients with bladder cancer (135).

Generally, the expression of TIM-3 and PD-1 on T cells is indicative of their impaired functionality (136). TIM-3 is overexpressed on CD8^+^ T cells in patients with SARS-CoV-2-induced disease (67), and its level is higher in cases with severe (vs. mild) disease, while it will become normalized in convalescent individuals (137). A higher number of functional (not exhausted) PD-1^+^ CD8^+^ T cells are also reported in patients with SARS-CoV-2 (138). IFN-γ-producing PD-1^+^ CD8^+^ T cells are active during the acute phase of viral infection (139). A point here is that PD-1 expression on the surface of CD8^+^ T cells is “transient” during the acute phase of viral infection in which upon viral clearance this receptor shows a downregulated expression profile (90). This is comparable with the “sustained” PD-1 expression on CD8^+^ T cells in patients with chronic infection or in cancer cases, rendering T-cell exhaustion or dysfunction (90, 140). PD(L)-1 is considered as a biomarker of poor prognosis only when PD-1 is overexpressed on antitumor immune cells, and simultaneously PD-L1 is highly expressed in the TME. This is important from a therapeutic standpoint in which a high expression of PD-1 on antitumor immune cells in a PD-L1^low^ tumor area is considered as a suppressor of tumor, whereas the cells placed in the PD-L1^high^ environment will lose their antitumor activity or may even take tumor-promoting function (99).

ICI seems to act at the cross-road between SARS-CoV-2-induced disease and cancer (141). ICI can be used for modifying the immune system in cancer patients with active SARS-CoV-2 (3). Cancer patients treated with PD(L)-1 blockade during the pre-infectious phase of SARS-CoV-2 are more resistant to the attack from the virus (142), and during the early phase of SARS-CoV-2 infection such therapy can contribute to the viral clearance (141). PD(L)-1 inhibitor therapy can thus provide a timeline therapeutic schedule for cancer patients (143), and restoring (or reactivation) of T-cell functionality mediated by such therapy can possibly be a winning step for beating SARS-CoV-2 infection (144). A recent study investigated the effects of chemo- or immunotherapy on the immune state in patients with gynecological malignancies who also had SARS-CoV-2 infection. Outcomes showed that in patients receiving chemotherapy no considerable changes occurred in the fraction and activity of B and T cells, but they mainly had neutropenia. By contrast, patients receiving PD-1 inhibitor therapy showed a massive rise in the cytotoxic scores for NK and T cells and that this therapy facilitated T-cell expansion in such cases (134). This is indicative of higher efficacy of immunotherapy over chemotherapy in cancer patients with active SARS-CoV-2.

A possible challenge for the use of ICI in cancer patients who also have active SARS-CoV-2 is a possible risk of experiencing cytokine release syndrome, which is a key contributor to the SARS-CoV-2-related mortality (3) and mediated *via* the impact of ICI in boosting T-cell migration toward the site(s) of infections (141). However, outcomes of a recent report attested augmented T-cell immunity in melanoma patients with SARS-CoV-2-induced disease treated with ICI and that such cases did not show exacerbation of inflammation (145). Pneumonitis occurring in patients with active SARS-CoV-2 is also presented in cases receiving ICI, which is seemingly indicative of a synergy in the manifestation of symptoms in such patients. However, pneumonitis is seen in 0.3% to 2% of patients receiving ICI, so it is not considered as a high-risk indicator for SARS-CoV-2 cases (3). Outcomes of a study by Luo and colleagues showed a safety profile for PD-1 inhibitor therapy in lung cancer patients who are also affected by SARS-CoV-2 (7). Results of a recent preliminary study also showed no rise in the rate of SARS-CoV-2-related mortality in melanoma patients receiving ICI therapy compared to the targeted therapy or treatment-naïve patients (146). These outcomes are indicative of the safety of PD(L)-1 inhibitor therapy in cancer patients with active SARS-CoV-2. A limited number of works in this area will ask for more future studies and gathering of more information in regard with the precise impact of ICI therapy in patients with SARS-CoV-2-induced disease.

### Key Note

PD(L)-1 blockade is presumably safe in cancer patients with SARS-CoV-2, and its application is beneficial for reducing the risk of attack from the virus.

## Factors Influencing CD8^+^ T-Cell Activity in SARS-CoV-2-Induced Disease and Cancer

### The Impact of Aging and Obesity

Most cancers are evolved in patients with ages over 60, the age range estimated to dominate more than 20% of the world’s society by year 2050, which is alarming as an economic burden related to healthcare provision (147). Outcomes of a recent systematic review showed more pronounced survival benefits related to the PD(L)-1 blockade among cancer patients aged <75 compared with cases ≥75 (148). The efficacy of the SARS-CoV-2 vaccination is also noticeably diminished with age, which is due presumably to a decline in responses from innate and adaptive immunity as a result of aging (149). CD8^+^ T cells are less frequent in aged HNSCC patients, and a high expression of PD-1 on peripheral T cells is reported in such population (150). Studies showed that CD8^+^ T cells are less frequent in SARS-CoV-2 patients aged >45 compared with cases with ages <45 (24), and that the cells are not detectable in patients over 80 (28). Besides, the basal pro-inflammatory state is higher in older individuals. These along with the diminished capability for mounting appropriate immune responses are reasons for the higher severity of condition in aged SARS-CoV-2 patients (151). Aging also causes higher accumulation of senescent immune cells (152), which is a reason for higher baseline inflammation in such populations (113) and their more vulnerability to SARS-CoV-2-induced diseases (152).

**Figure 6:**
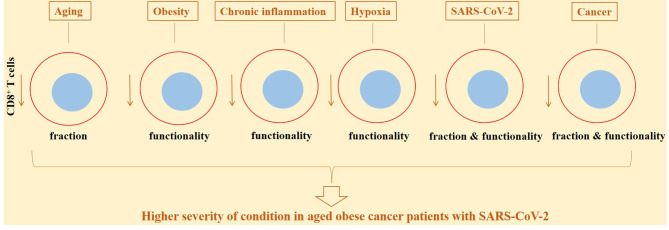
CD8^+^ T-cell diverse functionality and fraction in response to different conditions, in cancer patients and in cases with active SARS-CoV-2. The activity and fraction of CD8^+^ T cells are influenced from several conditions. Aging, obesity, chronic inflammation, and hypoxia are among the most important contributors in this context, rendering lower responses to anticancer therapies or reducing the efficacy of vaccination against SARS-CoV-2.

Obesity is an established risk factor of cancer development, which represents a global rise and poses a high cost over public health (153). Obesity is among preexisting conditions that cause patients to be prone to a more severe SARS-CoV-2 disease (154). A possibility of experiencing a severe disease is twice in obese individuals compared with normal-weight SARS-CoV-2 patients (155). Obesity alters the balance between innate and adaptive immunity. It links with a state of low-grade chronic inflammation (154). Chronic inflammation related to obesity alters immune functionality. The number of dysfunctional CD8^+^ T cells is increased with obesity (156). T-cell exhaustion is induced by cellular interactions occurring within adipose tissue (157). Generally, diets interfering with metabolic pathways are linked positively to the increased risk of carcinogenesis (158). CD8^+^ T cells show alterations in metabolic fitness in obese individuals. Such altered metabolic activity influences differentiation and effector activity of CD8^+^ T cells and their transition into memory cells (159). Impaired activity of CD8^+^ T cells also occurs in obese patients with SARS-CoV-2-induced disease, which is indicative of a more severe condition in such cases (156). A mechanism by which obesity contributes to the increased risk of tumorigenesis is by promoting constant upregulation of PD-1 on CD8^+^ T cells and their further dysfunction (160). Transition from PD-1^–^non-exhausted CD8^+^ T cells into PD-1^+^-exhausted CD8^+^ T cells is reported in breast cancer mice receiving high-fat diet (161). Taken together, it could be asserted that aging and obesity are key risk factors of cancer development, and aged or obese patients experience more severe disease upon exposure to SARS-CoV-2 due partly to the attenuated CD8^+^ T-cell activity (**Figure 6**).

### Key Note

CD8^+^ T cells are less frequent with aging, and their activity is diminished in obese individuals.

### Chronic Inflammation and Hypoxia

Chronic inflammation is a major risk factor of cancer. Conditions like chronic inflammation and autoimmunity account for about 15%–20% of all types of cancers. An organ with long-lasting inflammation is more prone to tumor development (162). Examples are numerous in this context, such as chronic obstructive pulmonary disease (COPD) (163), inflammatory bowel disease (IBD) (164), cirrhosis (165, 166), and chronic pancreatitis (167), which are the basis for the development of lung cancer, colorectal cancer, hepatocellular carcinoma (HCC), and pancreatic cancer. In regard to HCC, inflammatory-related events can cause chronic liver fibrosis and further chronic damages in this organ (168). A possible reason for an increase in the risk of tumorigenesis is the interrelation between chronic inflammation and chronic oxidative stress (17, 169) and the resulting impaired immune functionality and further tissue dysfunction (170). This is indicative of the virtue of using antioxidants, such as melatonin for modifying inflammatory and oxidative events in favor of immune activation against chronic injuries and cancer (171).

Chronic inflammation causes abnormal cytokine production (172), and patients with chronic inflammatory-related diseases are more prone to the SARS-CoV-2 infection. Diabetes, for instance, is a chronic inflammatory disease that affects cellular proliferation, differentiation, and function of innate and adaptive immunity (173). CD8^+^ T cells undergo exhaustion in the chronic inflammatory setting. CD8^+^ T cells served to trigger potent responses against viral infections for resolving infection. However, chronic infection is established when viruses are able to overcome this control. In fact, CD8^+^ T-cell exhaustion occurring in such conditions is a result of their constant responses to the long-lasting viral replication and the resulting continuous antigen stimulation (174). Chronic antigen stimulation drives CD8^+^ T-cell exhaustion, which further reduces their secretory cytokines. Finally, the cells undergo progressive loss of functionality (175) and apoptosis (174).

Hypoxia is another factor that is linked with tumorigenesis, tumor progression, and SARS-CoV-2 pathogenesis. Hypoxic areas are common in patients with solid tumors, which is due partly to the abnormal tumor vessels (176, 177). Hypoxia is induced by SARS-CoV-2 (178). It occurs as a response to severe respiratory dysfunction, which causes a discrepancy between uptake and consumption of O_2_ (179). HIFs are by-products of hypoxia that are stimulated within inflammatory tissues. There is a positive link between acute systemic hypoxia with mortality from severe SARS-CoV-2-induced diseases. SARS-CoV-2 promotes cytokine storm, particularly in the lung environment. HIF-1α functions as an inflammatory stimulant, mediated through provoking the transition of preexisting cytokine storm into a fulminant condition (178). Low O_2_ reduces antiviral responses from CD8^+^ T cells (180). Hypoxia reduces T-cell effector function through increasing the expression of co-inhibitors on CD8^+^ T cells (176). Hypoxic tumors generally show a lower fraction of CD8^+^ T cells. CD8^+^ T cells either avoid hypoxic regions or show reduced ability to expand in such areas (181, 182). Hypoxic tensions mainly influence antigen-experienced CD8^+^ T cells, namely, memory and effector cells (not naïve CD8^+^ T cells) (182). Exposure of exhaustive CD8^+^ T cells to hypoxic conditions will promote a terminal exhaustive state in such cells that are unable to recover their effector function after ICI therapy (183). Hypoxia induces the generation of reactive oxygen species (ROS) in T cells of patients with progressive (severe/critical) SARS-CoV-2-induced diseases, as compared with cases that recovered from critical diseases. High ROS accumulation and defective T-cell mitochondria are associated with severe SARS-CoV-2 diseases (184) (**Figure 6**).

### Key Note

Areas of chronic inflammation and hypoxia favor more dysfunctional CD8^+^ T cells in patients with SARS-CoV-2 induced diseases and cancer.

### Aberrant Angiogenesis and Abnormal Vasculature

SARS-CoV-2 in relation to hypoxia promotes vascular abnormality (a leaky architecture) (185). A leaky vasculature is also a common feature of cold cancers. The vascular endothelial growth factor (VEGF, also called VEGF-A) is a cytokine that vitally contributes to angiogenesis (186). The rate of VEGF expression is considerably higher in patients with SARS-CoV-2-induced disease compared with healthy individuals (187). High activity of VEGF promotes vascular permeability (186). Administration of the VEGF inhibitor bevacizumab in patients with severe SARS-CoV-2-induced disease significantly improved the PaO_2_/FiO_2_ ratio and O_2_ status (188). SARS-CoV-2 uses angiotensin-converting enzyme 2 (ACE2) as a receptor to attach the cellular membrane and enter the intracellular milieu. ACE2 is suppressed by SARS-CoV-2 upon cellular entry, and the activity of VEGF is inhibited by ACE2. The outcome of these inter-inhibitory effects is the high VEGF activity and acute lung injury (187).

Abnormal vasculature leads tumors toward metastasis. Metastasis is a main cause of cancer-related death, which accounts for over 90% of deaths from cancer (189). Abnormal tumor vasculature hampers effective infiltration of CD8^+^ T cells into the tumor area. Normalization of tumor vessels by strategies, such as M1 polarization (121), causes more infiltration of CD8^+^ T cells, thereby hampering immune escape and tumor metastasis (190). FASL expressed in tumor endothelial cells (ECs) is a barrier for CD8^+^ T-cell infiltration into the tumor area (186).

The expression of VEGF is upregulated in hypoxic conditions, and its upregulation reduces immune effector function. VEGF impedes DC maturation, thus causing CD8^+^ T-cell inactivation. VEGF induces PD-1 expression on CD8^+^ T cells and PD-L1 upregulation in the microenvironment of tumors like glioblastoma, thereby causing CD8^+^ T-cell exhaustion (191). VEGF-D is another cytokine in the VEGF family. VEGF-D contributes to the formation of lymphatic vessels (186). Upregulation of VEFG-D contributes to severe SARS-CoV-2-induced diseases (192). High VEGF-D expression is negatively related to the survival of patients with metastatic CRC (193). There is no direct evidence for the possible relation between VEGF-D with CD8^+^ T-cell activity in SARS-CoV-2 cancer patients, which requires focus in future studies.

### Key Note

High VEGF activity is presumably related to the CD8^+^ T-cell dysfunction in patients with a SARS-CoV-2-induced disease and cancer.

### Boosting CD8^+^ T-Cell Activity Against SARS-CoV-2-Induced Disease and Cancer

CD8^+^ T cells are without a doubt one of the most important cells of the immune system to act against SARS-CoV-2-induced disease and cancer, so their fraction and function are affected by a number of strategies for suppressing tumor development or vaccines against SARS-CoV-2. Evaluation of the fraction and functionality of these critical cells within circulation can, thus, be of prognostic, diagnostic, and therapeutic importance. The aim of new immunotherapeutic strategies is to target co-inhibitory receptors on T cells or improve their tumor recognition abilities and responses against tumor-specific antigens. In fact, responses to immunotherapy are evolved when T cells are directed more toward tumor neoantigens. Peptide-based vaccines are examples in this context. Short peptides directly bind to the HLA-I molecules and act as mounting MHC-I-restricted antigen-specific responses from CD8^+^ T cells. Long synthetic peptides must be presented by APCs to represent a T-cell response. Long peptide vaccination usually causes higher immunity compared with short peptides and induces responses from both CD4^+^ T-helper and CD8^+^ cytotoxic T cells when they are conjugated with effective adjuvant (194). T-cell expansion and strengthening of their antitumor reactivity are achieved after systemic administration of tumor-associated antigens (TAAs). Such approach is effective in patients receiving adoptive T-cell transfer (ACT) (195). The efficacy of antitumor T cells in patients receiving T-cell therapy can also be improved through improving T-cell stemness (196). CD8^+^ T cells in the TME are under exposure to hypoxic conditions, which poses a strong negative impact on their effector function. Thus, a suggested strategy is to modify HIF expression, HIF-2α in particular, in such cells in order to strengthen the adaptation of transferred T cells to the hypoxic conditions of such milieu (197). Outcomes of a recent study showed a positive link between breast cancer exposure to carcinogens with increased infiltration and strengthened antitumor activity of CD8^+^ T cells. Mice upon exposure to the 12-dimethylbenz[a]anthracene (DMBA) carcinogen showed cancer cell upregulation of chemokine (C–C motif) ligand 21 (CCL21) and strengthened antigen presentation activity. CCL21 activity was associated with higher tumor mutational burden (TMB) as well as strengthened T-cell immunity. CD8^+^ T cells were diminished after deletion of the CCL21 receptor, an outcome of which was severe liver and lung metastasis. The results of this study are indicative of the link between carcinogen exposure with stimulation of immune activating factors in cancer cells for potentiating the activity of CD8^+^ T cells against tumor metastasis (198). Cytokine-based therapy using agonists of the TNF receptor family has shown promising antitumor effects, mediated through strengthening the activity of CD8^+^ T cells. An example in this context is the CD137 agonist which shows antitumor activity against gastric cancer (199). Long-lasting responses from T cells can also be elicited using vaccine-based vectors. Cytomegalovirus, for instance, can be used as a vaccine vector for tumor-specific responses from CD8^+^ T cells (200). Strategies can also be expanded through strengthening the intra-tumoral infiltration of CD8^+^ T cells, as is known that cancer cells have developed several mechanisms to diminish homing of T cells and their access to the tumor tissue (201). Reshaping metabolic systems in CD8^+^ T cells is important for boosting their function in the TME that is generally under consistent nutrient and O_2_ deprivation. Strengthening fatty acid catabolism is a strategy to improve the antitumor capacity of CD8^+^ TILs. Such metabolically modified T cells presumably show higher antitumor potentials in patients receiving ICI therapy, such as PD-1 inhibitor drugs (202).

Strengthening CD8^+^ T-cell activity is not only effective for cancer but also promising against SARS-CoV-2-induced diseases. SARS-CoV-2 infection triggers cellular immune responses through CD8 overexpression and CTL hyperactivation (203). CD8^+^ T-cell responses are reported to be stimulated using SARS-CoV-2 vaccine boosters (204), and SARS-CoV-2 mRNA vaccines cause fast and stable mobilization of such cells (205). SARS-CoV-2-specific cellular and humoral responses to the inactivated virus vaccine CoronaVac immunization have also been attested (206).

## Conclusions

From what is discussed above, it can be concluded that the infiltration and activity of CD8^+^ T cells can be used as a biomarker of response in cancer patients with SARS-CoV-2-induced diseases, and their dysfunctionality is a dominant reason for a more severe condition. Persistent infection and sustained stimulation are factors associated with the exhaustive state in CD8^+^ T cells (10). In addition, unpredictable responses to immunotherapy are predictable in cancer patients due to the heterogeneity in TIL composition and phenotype (115). This along with the diminished responses from T cells to SARS-CoV-2 in cancer patients is linked with the severe infection and the dismal outcomes in SARS-CoV-2-infected cases with cancer (5). The severity of the condition is also affected from the type of cancer. In patients with lung cancer, for instance, more severe SARS-CoV-2 diseases are expected in comparison with gastrointestinal and breast cancers (207). Recovering the activity of such important cells is the key in the area of cancer therapy and in relation with the development of vaccines against SARS-CoV-2 and better outcomes of vaccination. In fact, the importance of the link between T-cell responses with milder disease will highlight the potential of considering non-spike proteins in SARS-CoV-2-based vaccination therapy (208). The acceleration or amplification of antiviral T cell immunity using ICIs can be a strategy for improving the efficacy of vaccination and establishing long-lasting immunity in cancer patients with active SARS-CoV-2 infection, as what was reported for melanoma patients (145).

## Author Contributions

Collection and revision of information, KM. Conceptualization, KM. Writing, original draft preparation, review, and editing, JM and KM. Both authors contributed to the article and approved the submitted version.

## Conflict of Interest

The authors declare that the research was conducted in the absence of any commercial or financial relationships that could be construed as a potential conflict of interest.

## Publisher’s Note

All claims expressed in this article are solely those of the authors and do not necessarily represent those of their affiliated organizations, or those of the publisher, the editors and the reviewers. Any product that may be evaluated in this article, or claim that may be made by its manufacturer, is not guaranteed or endorsed by the publisher.
